# Developmental expression of p97/VCP (Valosin-containing protein) and Jab1/CSN5 in the rat testis and epididymis

**DOI:** 10.1186/1477-7827-9-117

**Published:** 2011-08-19

**Authors:** Sevil Cayli, Seda Ocakli, Fikret Erdemir, Ufuk Tas, Huseyin Aslan, Tamer Yener, Zafer Karaca

**Affiliations:** 1 Department of Histology and Embryology, Faculty of Medicine, Gaziosmanpasa University, Tokat, Turkey; 2 Department of Urology, Faculty of Medicine, Gaziosmanpasa University, Tokat, Turkey; 3 Department of Anatomy, Faculty of Medicine, Gaziosmanpasa University, Tokat, Turkey; 4 Experimental Animal Center, Faculty of Medicine, Gaziosmanpasa University, Tokat, Turkey

## Abstract

**Background:**

The ubiquitin proteasome system (UPS) is a key player in regulating many cellular processes via proteasomal degradation of ubiquitinated proteins. Recently published data show that Jab1/CSN5 interacts with p97/VCP and controls the ubiquitination status of proteins bound to p97/VCP in mouse and human cells. However, coexpression of p97/VCP and Jab1/CSN5 in the developing rat testis and epididymis has not previously been studied.

**Methods:**

Testicular and epididymal tissues from 5-, 15-, 30-, and 60-day-old rats were examined by immunohistochemistry and Western blotting. Colocalisation of proteins was determined by immunofluorescence microscopy.

**Results:**

In the 5-day-old rat testis, p97/VCP and Jab1/CSN5 were specifically expressed in gonocytes. The expression of p97/VCP and Jab1/CSN5 significantly increased at day 15 and was found in spermatogonia, Sertoli cells and spermatocytes. In 30- and 60-day-old rat testes, p97/VCP indicated moderate to strong expression in Sertoli cells, spermatogonia, round and elongating spermatids. However, moderate to weak expression was observed in spermatocytes. Jab1/CSN5 showed strong expression in spermatogonia and spermatocytes, while relatively moderate expression was observed in round and elongating spermatids in 30- and 60-day-old rat testes. In contrast, in the epididymis, the expression of both proteins gradually increased from 5 to 60 days of age. After rats reached 2 weeks of age, the expression of both proteins was mostly restricted to the basal and principal cells of the caput epididymis.

**Conclusions:**

Our study suggests that p97/VCP and Jab1/CSN5 could be an important part of the UPS in the developing rat testis and epididymis and that both proteins may be involved in the regulation of spermatogenesis and epididymal epithelial functions.

## Background

The testis has the specific function of generating spermatozoa from precursors called spermatogonia after an intricate series of divisions [1]. This process takes place within the seminiferous epithelium, which is a complex structure composed of germ cells with radially oriented supporting cells termed Sertoli cells. In postnatal animals, spermatogenesis is initiated in the testes when gonocytes resume proliferation, migrate to the seminiferous tubule basal membrane and differentiate into spermatogonial stem cells [2]. The postnatal phase is divided into three main stages: 1) mitotic proliferation of spermatogonial stem cells and premeiotic differentiation of spermatogonia into diploid primary spermatocytes; 2) meiotic differentiation of primary spermatocytes into haploid early, round spermatids; and 3) spermiogenesis, a cellular and nuclear reorganisation process that differentiates spermatids into spermatozoa [3].

The epididymis provides an adequate environment for the final maturation of sperm [4-6]. During embryonic and postnatal development of the testis and epididymis, regulated proteolysis and organelle degradation are required [7-9]. The ubiquitin proteasome system (UPS) is a developmentally regulated and highly substrate-specific pathway for the removal of damaged and aberrant proteins. It is well known that the UPS fulfils necessary requirements for sperm cell differentiation inside the testicular seminiferous tubules and cell cycle control throughout spermatogenesis and fertilisation in adult males. Moreover, the ubiquitin-activating enzymes (E1), ubiquitin-conjugating enzymes (E2), ubiquitin ligase (E3) and some proteasomal subunits are expressed during spermatogenesis and postnatal testicular development [10-13].

In the male reproductive system, the UPS contributes to gamete quality control mechanisms, carrying out selective spermatogonial removal at the haploid phase of spermatogenesis [14], protein and organelle degradation during spermiogenesis [8,15], and the tagging defective spermatozoa with ubiquitin in the epididymis [7,16]. For these reasons, exploring the expression of proteins that are the components of the UPS may lead to advances in understanding the biology of the testis and epididymis.

In the ATP-dependent ubiquitin pathway, the attachment of ubiquitin to a target protein, referred to as ubiquitination, is carried out by E1, E2 and E3 [17]. The main purpose of ubiquitination is to deliver the ubiquitinated proteins to a cellular trash bin, a lysosome, an autophagosomal vacuole, or a 26S proteasome. Ubiquitinated proteins can either be transferred directly to the proteasome or indirectly transferred via p97/Valosin-containing protein (VCP), a member of the ATPase super-family associated with diverse cellular activities (AAA-ATPase). p97/VCP has been associated with a wide variety of essential cellular protein pathways, including nuclear envelope reconstruction, cell cycle regulation, Golgi reassembly, suppression of apoptosis, DNA damage responses, maturation of autophagosome and sperm capacitation [18-26]. In addition, during endoplasmic reticulum-associated degradation, p97/VCP dislodges ubiquitinated proteins from the endoplasmic reticulum (ER) and chaperones them to the cytosol for proteasomal degradation [27]. For ubiquitination of misfolded proteins in the ER, interaction with p97/VCP is required [28]. Moreover, it has recently been shown that the COP9 signalosome (CSN) interacts in an ATP-dependent manner with p97/VCP and controls the ubiquitination status of proteins bound to p97/VCP [29].

The CSN, which is involved in the ubiquitin/proteasome system, contains eight core subunits (CSN1-8), like the proteasome lid complex. CSN5 (also known as Jab1) facilitates the 26S proteasome-dependent degradation of several proteins, including p27Kip, luteinising hormone receptor (LHR), p53, oestrogen receptor, Smad4, Smad7, Id1, Id3, and IκBα [30-35]. The JAMM (JAB1/MPN/Mov34 metalloenzyme) domain present in Jab1/CSN5 exhibits deubiquitinase activity regulating ubiquitinlated protein sorting when associated with the CSN [36]. In addition to its role in the UPS, Jab1/CSN5 regulates many signalling pathways, such as transforming growth factor (TGF)-β signalling, cell proliferation, apoptosis and DNA repair [37].

Although it seems clear that the UPS is fundamentally required for the male reproductive system, there currently exists no information on the colocalisation patterns of p97/VCP and Jab1/CSN5 and their possible roles in the developing rat testis and epididymis. Therefore, the goal of this study was to assess the developmental expression of p97/VCP and Jab1/CSN5 and to show the cellular localisation of both complexes in rat testis and epididymis. For this reason, we analysed the expression of p97/VCP and Jab1/CSN5 in rat testis and epididymis during postnatal development using immunohistochemistry, immunofluorescence and Western blot techniques.

## Methods

### Animals and tissue preparation

After obtaining approval from the local ethics committee (2010-HADYEK-012), twenty-four male Wistar albino rats at postnatal ages of 5, 15, 30 and 60 days (six rats per group), i.e., corresponding to infantile (5 day), prepubertal (15 day), pubertal (30 day), and adult (60 day) periods, were obtained from the Gaziosmanpasa University Experimental Animal Research Laboratory. The rats were cared in the laboratory according to institutional guidelines and the *Guide for Care and Use of Laboratory Animals *of the National Research Council. All rats were observed for several days to ascertain their health status before sample collection. Pups were reared with their dams. They were maintained in a temperature-controlled room (20-23 °C) on a 12 h light/dark cycle with food (commercial rat chow) and fresh water available adlibitum. Six rats of the same age were killed by administration of an overdose of sodium pentobarbital (150 mg/kg, i.p.) before removal of the testis and epididymis. Epididymes were dissected and subdivided into three anatomical regions: the caput (CT), corpus (CS) and cauda (CA) epididymis. One testis and epididymis from each animal were fixed in Bouin's fluid for 12 h immediately upon collection, then dehydrated and embedded in paraffin for histochemistry and immunohistochemistry experiments (see below). The contralateral testis and epididymal regions from each animal were snap frozen in liquid nitrogen and strored at -80°C for Western blotting.

The definition of the stages and cell types with respect to the cycle of the seminiferous epithelium in adult testis was determined as previously published [38,39]. Germ cells were distinguished as being spermatogonia, spermatocytes, round and elongating spermatids based on their morphology and position in the seminiferous epithelium [40-42]. To spesificially identify mature Sertoli cells, p27^kip1 ^immunostaining was performed at days 30 and 60 testis and cytokeratin 18 immunostaining were used to determine immature Sertoli cells at days 15 [43,44].

### Immunohistochemistry

For immunohistochemical analysis, 5 μm-thick serial sections were collected on poly-L-lysine-coated slides (Sigma-Aldrich, St. Louis, MO, USA) and incubated overnight at 56 °C. Tissue sections were deparaffinised in xylene and rehydrated in a graded series of ethanol. Sections were then treated in a microwave oven in 10 mM citrate buffer, pH 6.0 and left to cool for 20 min. After three washes in phosphate buffered saline (PBS), endogenous peroxidase activity was quenched by 3% hydrogen peroxide in PBS for 20 min., and the sections were washed again three times in PBS. The sections were then incubated in a blocking serum (Ultra V Block, TP-060-HL; NeoMarker, Fremont, CA, USA) for 10 min. to block non-specific binding. Subsequently, sections were incubated overnight at 4°C with following primary antibodies: mouse monoclonal p97/VCP (MA3-004, 1: 500, Affinity BioReagent, USA), Jab1/CSN5 (sc-9074, 1: 200, Santa Cruz Tech, USA), p27^kip1 ^(sc-1641, 1: 100, Santa Cruz Tech, USA), p27^kip1 ^(sc-776, 1: 100, Santa Cruz Tech, USA), cytokeratin 18 (sc-58729, 1: 200, Santa Cruz Tech, USA), p97/VCP (sc-20799, 1: 200, Santa Cruz Tech, USA) and Jab1/CSN5 (ab495, 1: 250, Abcam, UK). The sections were then washed three times in PBS and incubated with biotinylated anti-mouse (BA-9200; 1:400 Dilution; Vector Laboratories, Burlingame, CA) and biotinylated anti-rabbit (BA-1000; 1:400 Dilution; Vector Laboratories) secondary antibodies for 45 min. at room temperature. After three washes with PBS, the antigen-antibody complexes were detected using a streptavidin-peroxidase complex (TP-060-HL; LabVision, Fremont, CA, USA) for 15 min., followed by three rinses with PBS. Bound peroxidase was developed with 3-amino-9-ethylcarbazol (AEC) (ScyTek Laboratories, USA) chromogen, and sections were counterstained with Mayer's hematoxylin (ScyTek Laboratories, Utah, USA) and mounted with Permount (Fisher Chemicals, Springfield, NJ, USA) on glass slides. For controls, sections were treated with the appropriate isotype of mouse IgG or rabbit IgG, depending on the primary antibody used, which was diluted to the same final protein concentration as the primary antibody. Photomicrographs were collected with a Leica microscope (Leica DM2500, Nussloch, Germany).

### Double immunofluorescence

To identify colocalisation of proteins, immunofluorescence was performed. Both primary antibodies (rabbit anti-Jab1/CSN5 and mouse anti-p97/VCP, mouse anti-p97/VCP and rabbit anti-p27^kip1^, mouse anti-Jab1/CSN5 and rabbit anti-p27^kip1^) were applied simultaneously and incubated overnight at 4°C. After rinsing in PBS, sections were incubated with FITC-conjugated goat anti-rabbit secondary antibody (at a 1:200 dilution, Millipore, USA) and rhodamine-conjugated goat anti-mouse secondary antibody (at a 1:500 dilution, sc-2092, Santa Cruz Biotechnology, USA) for 1 hour at room temperature. Thereafter, all sections were rinsed in PBS and nuclei were stained with DAPI (Invitrogen, Molecular Probes, D-1306). Fluorescence images were taken with a Nikon microscope (Nikon E600, Germany).

### H-SCORE analysis

Evaluation of the immunohistochemical labelling was performed using H-SCORE analyses as previously described [45]. The intensities of the p97/VCP and Jab1/CSN5 immunoreactivities were evaluated semi-quantitatively using the following intensity categories: 0 (no staining), 1+ (weak but detectable staining), 2+ (moderate or distinct staining), and 3+ (intense staining). For each tissue, an H-SCORE value was derived by calculating the sum of the percentages of cells that stained at each intensity category and multiplying that value by the weighted intensity of the staining using the Formula H-SCORE: ∑Pi(i+ l), where 'i' represents the intensity scores and 'Pi' is the corresponding percentage of cells. For each slide, five randomly selected areas were evaluated under a light microscope (40 x objective), and the percentage of cells exhibiting each intensity within these areas was determined at different times by two investigators who were not informed about the type and source of the tissues. The average score of both observers was used.

### SDS-PAGE and Western blot analyses

Total testicular and epididymal proteins were extracted using modified RIPA buffer (1% NP-40; 0.25% sodium deoxycholate; 150 mM NaCl; 1 mM EDTA; 1 mM PMSF; 1 mg/ml each of aprotinin, leupeptin, and pepstatin; 1 mM Na3VO4; and 1 mM NaF in 50 mM Tris-Cl, pH 7.4) and quantitated using the Bradford procedure (Bio-Rad, Hercules, CA). Then, 40-μg samples were separated by 8% SDS-PAGE and electroblotted onto a nitrocellulose membrane (Bio-Rad Laboratories). The membrane was blocked with 5% non-fat dry milk in TBS containing 0.1% Tween 20 (TBS-T) for 1 h to reduce non-specific binding. Subsequently, the membrane was incubated for 1 h with primary antibodies against p97/VCP (MA3-004, Affinity BioReagent, USA, 1:10,000 in 5% non-fat dry milk), Jab1/CSN5 (sc-9074, Santa Cruz Biotechnology, 1:500 in 5% non-fat dry milk) and β-actin (sc-47778, C4; Santa Cruz Biotechnology Inc., 0.05 mg/ml in 5% non-fat dry milk). The membrane was washed with TBS-T for 1 h and incubated with horseradish peroxidase-conjugated anti-mouse and anti-rabbit secondary antibodies (Vector Laboratories) diluted in 5% non-fat dry milk in TBS-T. Bound secondary antibodies were visualised using an enhanced chemiluminescence substrate (GE Healthcare). Immunoblot bands for p97/VCP, Jab1/CSN5 and β-actin were quantified using an Alpha DigiDoc 1000 gel documentation unit (Alpha Innotech Corporation, CA, USA). The optical density (OD) values for the p97/VCP and JAb1/CSN5 bands were divided by the OD values of cognate β-actin bands to normalise the OD values for loading differences.

### Statistical analysis

The Mann-Whitney *U*-test was employed for comparison of independent groups of samples, and Kruskall-Wallis analysis with the Dunn posthoc test was performed for multiple comparisons of independent groups of samples. A *P*-value of less than 0.05 was considered to indicate a statistically significant difference. Statistical calculations were performed using SigmaStat for Windows, version 3.5 (Jandel Scientific Corp., San Rafael, CA).

## Results

### Cellular localisation of p97/VCP and Jab1/CSN5 in the developing rat testis

**Figure 1:**
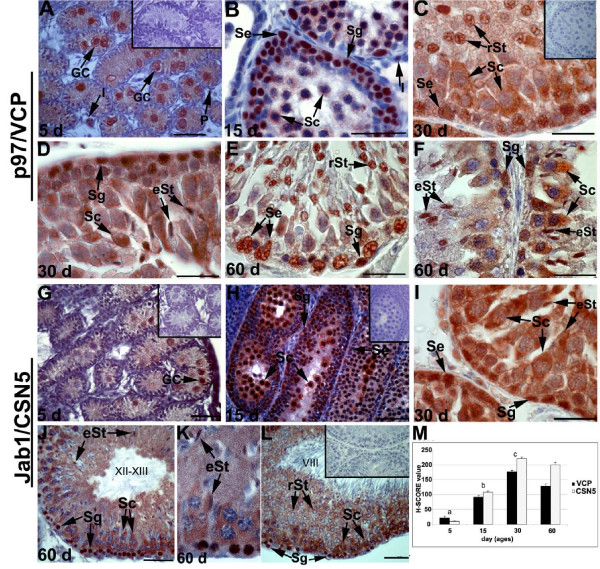
**Localisation of p97/VCP and Jab1/CSN5 in the developing rat testis**. Immunohistochemistry was used to show the cellular localisation of p97/VCP and Jab1/CSN5 in the testis at days 5 (A, G), 15 (B, H), 30 (C, D, I), and 60 (E, F, J, K, L) after birth. The negative controls (A, C, G, H, L, inserts). A: Positive staining of p97/VCP expression was demonstrated in the nuclear and cytoplasmic regions of gonocytes (GC) at day 5. Interstitial cells (I) show weak immunoreactivity, and peritubuler cells (P) present no immunoreactivity. B: p97/VCP is highly expressed in spermatogonia (Sg), spermatocytes (Sc) and Sertoli cells (Se) at day 15. Some I show moderate immunostaining for p97/VCP. C, D: Elongating (eSt) and round (rSt) spermatids, Se and Sg show moderate to strong immunostaining for p97/VCP, while cytoplasm of Sc are moderately labelled with p97/VCP. E, F: In the adult testis, p97/VCP is weakly to moderately expressed in the cytoplasm of Sc, whereas Se, Sg, rSt and eSt are moderate to strong immunopositive for p97/VCP. G: Moderate Jab1/CSN5 expression is seen at day 5 in GC. H: Jab1/CSN5 is strongly expressed in Sg and Sc while Se exhibit moderate expression at day 15. I: Sg, Sc, eSt and Se show moderate to strong immunostaining for Jab1/CSN5. J-L: In the adult testis, Jab1/CSN5 is strongly expressed in Sg and Sc, while rSt and eSt show weak to moderate immunostaining for Jab1/CSN5. Roman numerals indicate stages of the seminiferous epithelial cycle. M: H-SCORE of the p97/VCP and Jab1/CSN5 immunostaining intensities in the developing rat testis. The data are represented as the means ± SEM. a: p < 0.05, day 5 vs. days 15, 30 and 60, b: day 15 vs. day 5 and day 30, c: day 30 vs. days 5 and 15. Scale bars: 50 μm.

Immunohistochemical analysis of 5-day-old rat testes revealed that p97/VCP was mainly localised to nuclear and cytoplasmic regions of gonocytes in the lumen of the seminiferous tubules (Figure 1A). No immunoreactivity was detected in somatic cells, though some interstitial cells showed immunopositivity for p97/VCP (Figure 1A). In the 15-day-old rat testis, spermatogonia at the basal membrane, Sertoli cells and spermatocytes were positive for p97/VCP (Figure 1B, Table 1). Additionally, interstitial cells showed weak to moderate immunostaining for p97/VCP at day 15 (Figure 1B). The staining intensity and the number of cells positively stained for p97/VCP significantly increased by day 15, coinciding with the appearance of leptotene spermatocytes [39] (Figure 1B and 1M). The other cell types present in the 30-day-old rat testis, specifically round and elongating spermatids, also expressed p97/VCP (Figure 1C and 1D). Additionally, supporting Sertoli and peritubuler myoid cells, interstitial cells showed relatively moderate expression of p97/VCP, reaching the highest expression level at day 30 (Figure 1C-D and 1M). In adult rat (60 day) testis, p97/VCP indicated moderate to strong expression in the Sertoli cells, spermatogonia, round and elongating spermatids; however, spermatocytes showed moderate to weak expression of p97/VCP (Figure 1E-F, Table 1). The staining intensity and the number of p97/VCP-positive cells were found to be the same in all stages of seminiferous tubules in the adult rat testis. There was no immunoreactivity observed on negative control slides that were treated with the isotype mouse antibody instead of the p97/VCP primary antibody at the same final concentration (Figure 1A, 1C, inserts).

**Table 1:** Localisation of Jab1/CSN5 and p97/VCP in rat testis

	GC		Sg		Sc		rSt		eSt		Se		I	
**Postnatal Day**	**Jab1**	**VCP**	**Jab1**	**VCP**	**Jab1**	**VCP**	**Jab1**	**VCP**	**Jab1**	**VCP**	**Jab1**	**VCP**	**Jab1**	**VCP**

5	++	++	--	--	--	--	--	--	--	--	--	--	--	+
15	--	--	+++	+++	+++	++	--	--	--	--	++	++	+	+
30	--	--	+++	+++	++	++	++	++	++	++	++	++	++	++
60	--	--	+++	+++	++	+	+	++	++	++	+	+++	+	+

Semiquantitive evaluation of Jab1/CSN5 and p97/VCP immunoreactive cells in the developing rat testis. GC: Gonocytes, Sg: Spermatogonia, Sc: Spermatocyte, rSt: Round spermatid, eSt: Elongating spermatid, Se: Sertoli cell, I: Interstitial cells.

weak immunoreactivity: +, moderate immunoreactivity: ++, strong immunoreactivity: +++

In the 5-day-old rat testis, Jab1/CSN5 showed moderate immunolabelling in gonocytes (Figure 1G). The staining intensity and the number of Jab1/CSN5-positivite cells were significantly increased in the 15-day-old rat testis compared to the 5-day-old rat testis (Figure 1H and 1M). In the 15-day-old rat testis, Jab1/CSN5 localised strongly in spermatogonia and spermatocytes, but Sertoli cells presented only moderate to weak immunostaining (Figure 1H, and Table 1). In the 30-day-old rat testis, spermatogonia, spermatocytes, round and elongating spermatids, and Sertoli and interstitial cells were positively labelled with Jab1/CSN5, presenting the highest expression level observed (Figure 1I, Table 1).

In the 60-day-old rat testis, Jab1/CSN5 showed stronger nuclear staining in spermatogonia and moderate cytoplasmic staining in spermatocytes at stages 8-12 (Figure 1J-L). However, the acrosomes of round spermatids at stages 6-8 and the heads of elongating spermatids at stages 1-5 and 9-14 displayed relatively weak immunostaining for Jab1/CSN5 (Figure 1K, 1L). A few interstitial cells and Sertoli cells presented weak labelling for Jab1/CSN5. No immunoreactivity was detected on control slides (Figure 1G, 1H and 1L, inserts). According to H-SCORE analysis, the expression of both Jab1/CSN5 and p97/VCP significantly increased from day 5 to day 30 in the testis, whereas no significant differences were found between day 30 and day 60 testes (Figure 1M).

**Figure 2:**
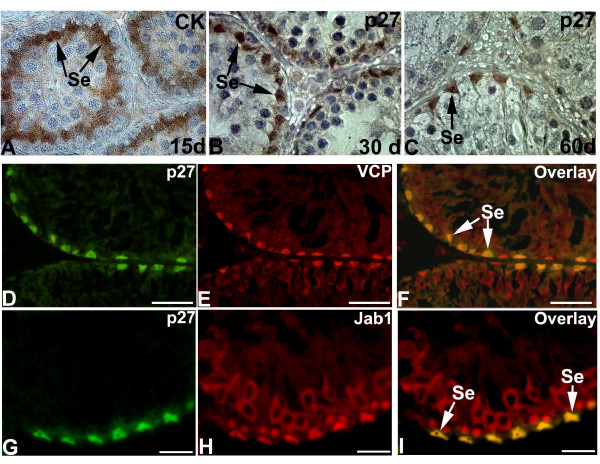
**Immunohistochemical analysis of Sertoli cells at day 5 (A), 15 (B), 60 (C) and colocalisation of p97/VCP - p27^kip1 ^(D-F) and Jab1/CSN5 - p27^kip1 ^(G-I) in rat testis**. Cytokeratin 18 (CK) are detected in immature Sertoli cells (Se) at day 15 (A) and p27^kip1 ^is observed in mature Sertoli cells (Se) of day 30 and 60 (B, C) in rat testis. Mature Sertoli cells (Se) are immunopositive for p27^kip1 ^(green, D, G), p97/VCP (red, E) and Jab1/CSN5 (red, H), resulting in a yellow color (overlay, F, I). Scale bars: 25 μm.

Immunoexpression of immature and mature Sertoli cell markers such as cytokeratin 18 and p27^kip1 ^was used to confirm whether p97/VCP and Jab1/CSN5 was expressed in Sertoli cells at days 15, 30 and 60 days of rat testis (Figure 2A-F). At days 15, Sertoli cells were immunopositive for cytokeratin 18 (Figure 2A). At days 30 and 60, p27^kip1 ^was found to be expressed in the mature Sertoli cells (Figure 2B and 2C). Moreover, p27^kip1 ^was colocalised with p97/VCP (Figure 2D-F) and Jab1/CSN5 (Figure 2G-I) in the mature Sertoli cells.

### Cellular localisation of p97/VCP and Jab1/CSN5 in the developing rat epididymis

**Figure 3:**
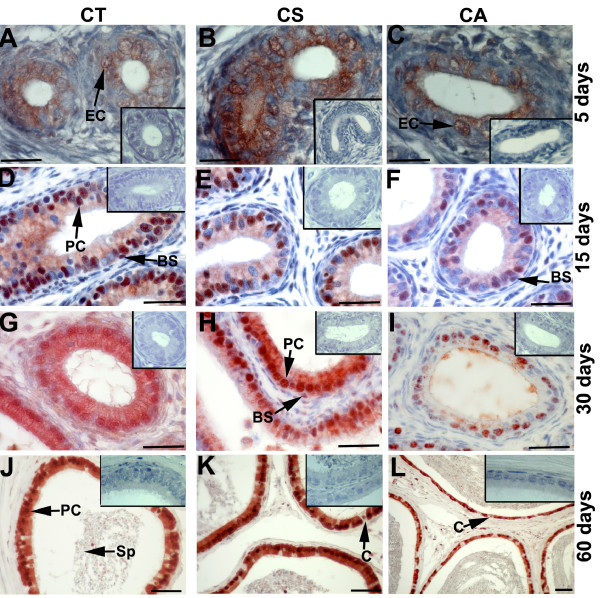
**Distribution of p97/VCP in different regions of the rat epididymis during postnatal development**. p97/VCP immunopositivity is detected in the CT, CS and CA of the epididymis at days 5 **(A, B, C)**, 15 **(D, E, F)**, 30 **(G, H, I) **and 60 **(J, K, L)**. p97/VCP expression is seen in the epithelial cells (EC) of the CT, CS and CA epididymis at day 5 (A, B, C). No significant staining is observed in the negative controls (at the corner of panels A-L). p97/VCP is localised both in the basal (BS) and principal (PC) cells of the epididymis at days 15, 30 and 60 (D-L). Of note, p97/VCP is also expressed in the apical layer of the cytoplasm in all epididymal regions at days 30 and 60 (G-L). Immunopositivity for p97/VCP is detected both in the principal cells and basal epithelial cells, while clear cells (C) show no immunoreactivity at day 60 (J-L). Spermatozoa (Sp) are weakly immunopositive for p97/VCP. CT: caput, CS: corpus, CA: cauda. Scale bars: 25 μm (A-C); scale bars: 50 μm (D-L).

In the 5-day-old rat epididymis, p97/VCP immunostaining was found in the CT, CS and CA epididymis. Epithelial cells of epididymis were positively labelled for p97/VCP (Figure 3A-C). p97/VCP immunoreactivity showed no uniformity in different regions of the epididymis, and high levels of expression were observed in CS at day 5 (Figure 3B). However, p97/VCP expression was higher in the CT and CS epididymis compared to the CA epididymis from day 15 to day 60 (Figure 3D-L, Table 2). p97/VCP immunostaining was seen in both the basal and principal cells of the adult rat epididymis (Figure 3J-L, Table 2). Moreover, in the lumen of the epididymis, spermatozoa presented weak staining for p97/VCP at day 60 (Figure 3L).

**Table 2:** Localisation of Jab1/CSN5 and p97/VCP in rat epididymis

	Caput				Corpus				Cauda			
	**PR**		**BS**		**PR**		**BS**		**PR**		**BS**	
**Postnatal Day**	**Jab1**	**VCP**	**Jab1**	**VCP**	**Jab1**	**VCP**	**Jab1**	**VCP**	**Jab1**	**VCP**	**Jab1**	**VCP**

5	+	+	--	+	+	++	--	+	+	+	--	+
15	+	+	+	++	++	++	+	++	+	+	+	+
30	+++	+++	++	++	++	+++	++	++	++	++	+	+
60	+++	+++	+++	+++	+++	+++	+++	+++	++	++	++	++

Semiquantitive evaluation of Jab1/CSN5 and p97/VCP immunoreactive cells in the developing rat epididymis. PR: Principal cell, BS: Basal cell.

weak immunoreactivity: +, moderate immunoreactivity: ++, strong immunoreactivity: +++

**Figure 4:**
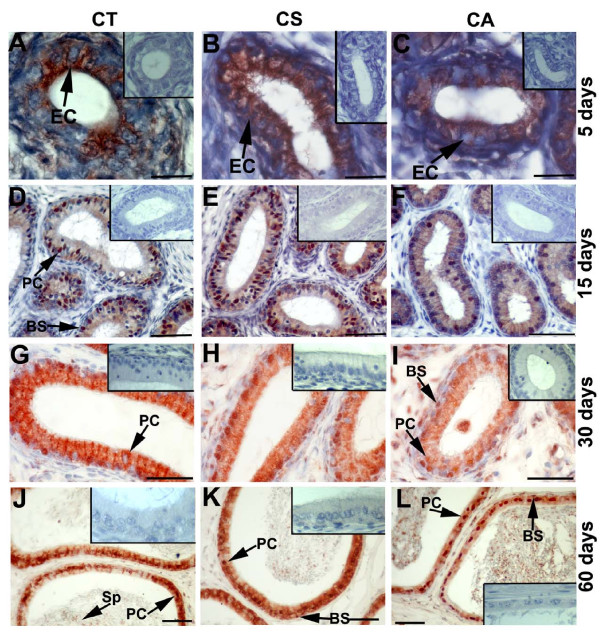
**Distribution of Jab1/CSN5 in different regions of the rat epididymis during postnatal development**. Jab1/CSN5 immunopositivity is detected in the CT, CS and CA at days 5 **(A, B, C)**, 15 **(D, E, F)**, 30 **(G, H, I) **and 60 **(J, K, L)**. Jab1/CSN5 is found in the cytoplasm of epithelial cells (EC) of the epididymis at day 5 (A, B, C). Both the basal (BS) and principal (PC) cells of the CT, CS and CA epididymis are found to be immunopositive for Jab1/CSN5 at days 15, 30 and 60 (D-L). In addition to the nuclear region of epithelial cells (EC), Jab1/CSN5 is highly expressed in the cytoplasm in the CT and CS epididymis at days 30 (G-I) and 60 (J-L). Spermatozoa (Sp) are weakly immunopositive for Jab1/CSN5 at day 60 (J-L). No significant staining is observed in the negative controls (at the upper corner of panels A-L). CT: caput, CS: corpus, CA: cauda. Scale bars: 25 μm (A-C); scale bars: 50 μm (D-L).

In 5-day-old rat epididymes, Jab1/CSN5 immunopositivity was detected in the CT, CS and CA regions (Figure 4A-C). Although some epithelial cells showed no immunoreactivity, Jab1/CSN5 was expressed in both the principal and basal cells at day 15, 30 and 60 in the epididymis (Figure 4D-L). On days 5 and 15, Jab1/CSN5 expression was relatively higher in the CS epididymis (Figure 4B-E); however, the CT epididymis presented increased expression in day 30 and 60 testes (Figure 4G, 4J). Spermatozoa showed weak immunostaining in the lumen of day 60 epididymes (Figure 4J-L).

### Colocalisation of Jab1/CSN5 and p97/VCP in the developing rat testis and epididymis

**Figure 5:**
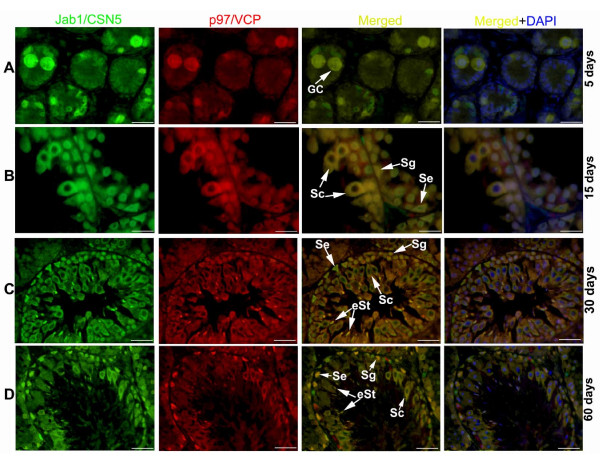
**Double immunohistochemistry representing the colocalisation of p97/VCP and Jab1/CSN5 in the developing rat testis (A-D)**. Detection of Jab1/CSN5 (green), p97/VCP (red), and double-stained (yellow) positive cells merged with DAPI nuclear staining on day 5 (A), day 15 (B), day 30 (C) and day 60 (D). **A: **Day 5 gonocytes (GC) are double labelled with p97/VCP and Jab1/CSN5. **B: **Double-labelled spermatocytes (Sc), spermatogonia (Sg) and Sertoli cells (Se) are shown on day 15, and the nuclear regions of some spermatocytes are only positive for Jab1/CSN5. **C: **Jab1/CSN5 and p97/VCP positivity (yellow) is observed in spermatocytes (Sc), spermatogonia (Sg), Sertoli cells (Se) and elongating spermatids (eSt) on day 30. **D: **Double staining (yellow) is detected in the cytoplasm of spermatocytes (Sc), the nuclear region of spermatogonia (Sg) and elongating spermatids (eSt) and Sertoli cells (Se) in the 60-day-old rat testis. Scale bars: 25 μm.

**Figure 6:**
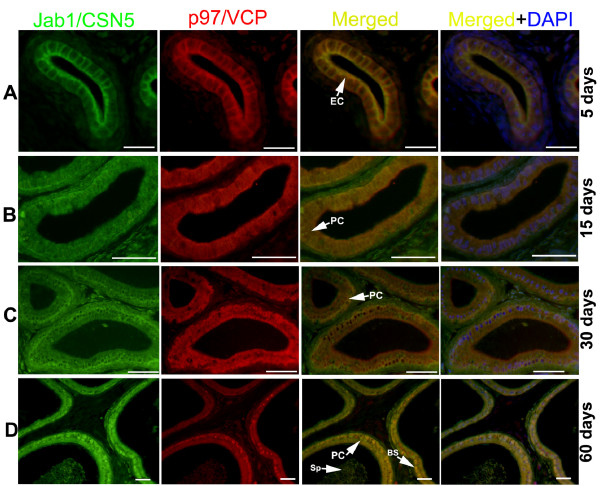
**Colocalisations of Jab1/CSN5 and p97/VCP in the developing rat epididymis (A-D)**. Detection of Jab1/CSN5 (green), p97/VCP (red), and double (yellow) positive cells merged with DAPI nuclear staining on day 5 (A), 15 (B), 30 (C) and 60 (D) CS epididymis. Both the basal (BS) and principal cells (PC) of CS epididymis are immunopositive for Jab1/CSN5 (green) and for p97/VCP (red), resulting a yellow color. Spermatozoa (Sp) are weakly immunopositive for Jab1/CSN5 and p97/VCP on day 60. Scale bars: 25 μm (A), 50 μm (B-D).

To determine Jab1/CSN5 and p97/VCP colocalisation, double immunofluorescence was performed in the testicular (Figure 5) and epididymal tissues (Figure 6). In the 5-day-old rat testis, Jab1/CSN5 and p97/VCP were found to be colocalised in gonocytes (Figure 5A). In the 15-day-old rat testis, p97/VCP immunostaining was overlapped with Jab1/CSN5 in spermatogonia, spermatocytes and Sertoli cells (Figure 5B). Although p97/VCP and Jab1/CSN5 staining intensities indicated differences in day 30, cytoplasm of spermatocytes, Sertoli cells, spermatogonia, round (rSt) and elongating (eSt) spermatids were found to be immunopositive for Jab1/CSN5 and p97/VCP (Figure 5C). In the 60-day-old rat testis, spermatogonia, spermatocytes, Sertoli cells and elongating spermatids were found to be double immunopositive for Jab1/CSN5 and p97/VCP, although some Sertoli cells and spermatogonia showed only Jab1/CSN5 or p97/VCP immunpositivity (Figure 5D).

In the 5-day-old rat epididymis, Jab1/CSN5 and p97/VCP were colocalised in the cytoplasm of the epithelial cells of corpus epididymis (Figure 6A). In the 15, 30 and 60-day-old rat epididymis, both proteins were found to colocalise in the nuclear and cytoplasmic regions (Figure 6B-D). Overall, the expression of Jab1/CSN5 and p97/VCP was gradually increased from 5 to 60 days of epididymis.

### Changes in protein expression levels of p97/VCP and Jab1/CSN5 in the developing rat testis and epididymis

**Figure 7:**
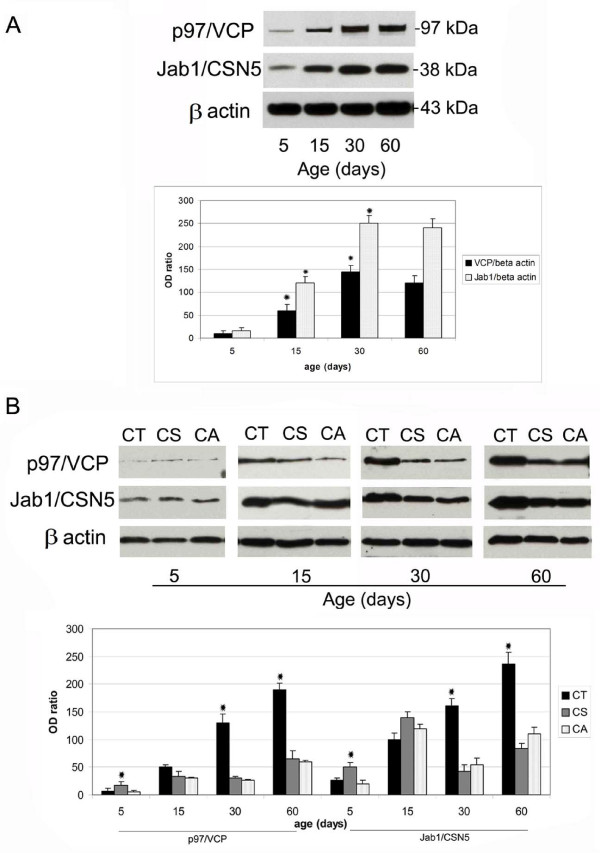
**Western blot analysis of p97/VCP and Jab1/CSN5 in the developing rat testis (A) and epididymis (B)**. **A: **p97/VCP (97 kDa) and Jab1/CSN5 (38 kDa) were detected by western blotting. β-actin (43 kDa) was used as loading control. Immunoblot bands were quantified by an optical densitometer. The OD (optical density) values of the p97/VCP and Jab1/CSN5 bands were normalised to the OD values of β-actin bands. The data in the graphs are presented as the means ± SEM. The expression of p97/VCP and Jab1/CSN5 is significantly increased from days 5 to 30 in the testis (Asterisks indicate, p < 0.05); however, no differences are observed between days 30 and 60. **B: **p97/VCP and Jab1/CSN5 expression levels are significantly higher in the CT epididymis than the CS and CA epididymis at days 30 and 60. Asterisks indicate p < 0.05, days 5 CS vs. days 5 CA and CT epididymis, days 30 and 60 CT vs. days 30 and 60 CA and CS epididymis. CT: caput, CS: corpus, CA: cauda.

In agreement with immunohistochemistry, Western blot results confirmed the presence of p97/VCP and Jab1/CSN5 in the developing rat testis and epididymis. Western blot analyses revealed a specific band at 97 kDa for p97/VCP and at 38 kDa for Jab1/CSN5. The intensity of both bands was quantified and normalised for the intensity of β-actin controls (Figure 7A-B, lower panels). The expression level of p97/VCP was low on day 5 of testis and epididymis but increased and reached a peak on day 30. However, no significant changes were observed between 30 and 60-day-old rat testis for p97/VCP expression (Figure 7A). Jab1/CSN5 expression was also increased gradually but reached the peak on day 30 in testis and on day 60 in the epididymis (Figure 7A-B). In accordance with immunohistocehemical results, p97/VCP and Jab1/CSN5 expressions were significantly higher in the CT epididymis compared to the CS and CA epididymis at day 30 and day 60 epididymis (Figure 7B, lower panel).

## Discussion

To our knowledge, this is the first study demonstrating coexpression of the UPS components p97/VCP and Jab1/CSN5 during postnatal development of the rat testis and epididymis using immunohistochemistry, immunofluorescence and Western blotting. Our results indicate that p97/VCP expression overlapped with Jab1/CSN5 expression in gonocytes, spermatogonia, spermatocytes, Sertoli cells, spermatids and epididymal epithelial cells in the 5-, 15-, 30- and 60-day-old rat testis and epididymis.

To define specific sites where p97/VCP and Jab1/CSN5 expression might be important, we explored cell-specific expression of p97/VCP and Jab1/CSN5 in the developing rat testis and epididymis. In the 5-day-old rat testis, we observed that p97/VCP and Jab1/CSN5 were specifically expressed in gonocytes and that the expression levels of these proteins were significantly lower compared to what was seen at other ages. The low levels of p97/VCP and Jab1/CSN5 expression in the neonatal testis suggest that neither protein has a major role during very early postnatal stages. However, from day 5 after birth to day 30, the levels of the p97/VCP and Jab1/CSN5 expression significantly increased. It has previously been shown that the period from 4 days after birth to 6 weeks of age corresponds to a period of rapid cell proliferation and growth of the rat testis and epididymis [3]. Therefore, the gradual increase in the expression of p97/VCP and Jab1/CSN5 coincides with testicular growth, suggesting that the p97/VCP and Jab1/CSN5 proteins may play important roles in cell proliferation in the testis and epididymis. In fact, it is well known that Jab1/CSN5 plays an essential role in cell growth and that strong expression of Jab1/CSN5 is associated with accelerated proliferation [46].

During postnatal development, the rat testis is composed of several developmental stages [39]. The rat testis at postnatal days 0-5 only contains gonocytes and somatic cells. By days 6-7, spermatogonia appear. By day 13-23, spermatocytes are present, and round spermatids are first observed by day 24-25. At postnatal day 30, elongating spermatids are seen, and by day 36, elongated spermatozoa can be found. In the present study, p97/VCP and Jab1/CSN5 were found to exhibit notable expression in the rat testis and to be present at virtually every phase of germ cell development and maturation. Therefore, these proteins might have important roles in the developing rat testis and epididymis. For example, as shown by the staining presented in Figure 1 and Figure 5, p97/VCP and Jab1/CSN5 localised in round and elongating spermatids, where the proteins are included in the developing acrosome and the sperm tail. Additionally, both proteins were expressed in maturing spermatocytes and spermatogonia, suggesting that these proteins may also be critical for normal spermatocyte development. Moreover, p97/VCP and Jab1/CSN5 immunoreactivity appeared in the Sertoli cells of juvenile animals (15- and 30-day-old rats) and mature rats (60-day-old rats).

Ultrastructural investigations of Sertoli cell have revealed that numerous phagosomes are located in the cytoplasm of these cells, which indicates the occurrence of phagocytic activity [43]. These authors indicated that Sertoli cells are highly involved in the digestion of germ cells that degenerate during spermatogenesis and of lobules of residual spermatid cytoplasm left during spermiation. Some researchers have also reported that Sertoli cells might be involved in the exchange and elimination of excessive sperm cell substances [47,48]. Recently, p97/VCP was found to be essential for autophagosome maturation, suggesting that p97/VCP might be selectively required for autophagic degradation of ubiquitinated substrates [24,25]. Based on the localisation of the p97/VCP in immature and mature Sertoli cells, our results support previous observations, and we suggest that p97/VCP may be necessary for the processing of ubiqutinylated and misfolded proteins from larger protein complexes or membranes in Sertoli cells. Further functional studies will be required to clarify the physiological role of p97/VCP in Sertoli cells.

In the present study, it was found that the expression of p97/VCP and Jab1/CSN5 gradually increased during postnatal development of the rat epididymis. Moreover, both proteins were observed in the epididymal epithelium and produced by all regions of the epididymis but were highly expressed in the CT and CS epididymis. These findings also support the idea that p97/VCP and Jab1/CSN5 probably contribute to the sperm maturation process in the epididymis.

There are number of pieces of evidence that removal and degradation of defective spermatozoa occurs during epididymal passage [8,16,49]. However, it is not clear how the defective spermatozoa are removed or what components of the UPS contribute proteasomal proteolysis in the epididymis. One possibility that arises from the present study is that Jab1/CSN5 together with p97/VCP may act as an important mediator during the proteasomal degradation of defective spermatozoa. It is well known that the JAMM domain of Jab1/CSN5 has deubiquitinase activity when associated with the CSN [36]. This activity of Jab1/CSN5 might be critical for protein degradation in the epididymis. Furthermore, it was previously shown when the CSN was inactivated by knockdown of Jab1/CSN5, the amount of polyubiquitinated proteins bound to p97/VCP increased, indicating that the CSN is required for proper processing of substrate proteins bound to p97/VCP [29]. Clearly, p97/VCP and Jab1/CSN5 may also be involved in the proper processing of polyubiquitinated substrates in the epididymis. Moreover, the colocalisation of p97/VCP and Jab1/CSN5 in 5-, 15-, and 30-day-old rat testes and epididymes also supports the previous findings indicating that Jab1/CSN5 may regulate the ubiquitination status of proteins bound to p97/VCP.

## Conclusions

Here, we report the developmental expression and colocalisation of the UPS components p97/VCP and Jab1/CSN5 in the rat testis and epididymis. Further research might contribute to the clarification of the exact functions of these proteins in the development of the rat testis and epididymis.

## Competing interests

The authors declare that they have no competing interests.

## Authors' contributions

SC and SO performed the major part of the histological analysis and wrote the manuscript. FE, TY, ZK, UT and HA participated in the study design and the analysis. All authors read and approved the final manuscript.
